# Inhibition of CUB and sushi multiple domains 1 (CSMD1) expression by miRNA-190a-3p enhances hypertrophic scar-derived fibroblast migration in vitro

**DOI:** 10.1186/s12864-021-07920-8

**Published:** 2021-08-12

**Authors:** Shuchen Gu, Xin Huang, Xiangwen Xu, Yunhan Liu, Yimin Khoong, Zewei Zhang, Haizhou Li, Yashan Gao, Tao Zan

**Affiliations:** Department of Plastic and Reconstructive Surgery, Shanghai Ninth People’s Hospital, Shanghai JiaoTong University School of Medicine, 639 Zhizaoju Road, Shanghai, 200011 P. R. China

**Keywords:** Hypertrophic scar, CSMD1, microRNA-190a-3p

## Abstract

**Background:**

Hypertrophic scar (HTS) is a fibroproliferative skin disorder characterized by excessive cell proliferation, migration, and extracellular matrix (ECM) deposition. The CUB and Sushi multiple domains 1 (CSMD1) has previously been identified as the key regulatory gene of hypertrophic scar by a large sample GWAS study. However, further research has not yet been conducted to verify this finding in other HTS patients and to determine the underlying mechanism.

**Results:**

In this study, we verified that CSMD1 was downregulated in both HTS tissue and HTS-derived fibroblasts. The knockdown of CSMD1 resulted in enhanced migration and fibronectin1 (FN1) secretion in fibroblasts in vitro. In addition, the upstream and downstream regulatory mechanisms of CSMD1 were also investigated through microRNA (miRNA) databases screening and RNA-sequencing (RNA-seq) respectively. The screening of four common microRNA (miRNA) databases suggested that miR-190a-3p binds to the CSMD1 and may regulate its expression. We confirmed that miR-190a-3p directly targeted the CSMD1–3′-UTR using luciferase reporter assays. Furthermore, the overexpression of miR-190a-3p showed promotion of migratory activity and FN1 secretion in fibroblasts, resembling the effect of CSMD1 knockdown; whereas the knockdown of miR-190a-3p exerted the opposite effect. Finally, transcriptomic analysis showed activation of Janus kinase-signal transducer and activator of transcription (JAK/STAT) signaling pathway in the CSMD1 knockdown fibroblasts.

**Conclusions:**

This study has validated the conclusions of the previous GWAS study conducted in Chinese population. In vitro experiments have provided further evidence on the function of CSMD1 in the development of HTS, and have also revealed the underlying upstream and downstream regulating mechanisms. Additionally, the JAK/STAT signaling pathway identified using RNA-seq might provide a potential treatment approach, especially for HTS.

**Supplementary Information:**

The online version contains supplementary material available at 10.1186/s12864-021-07920-8.

## Background

Hypertrophic scar (HTS) is a frequently encountered fibroproliferative skin disorder characterized by excessive cell proliferation, migration, and extracellular matrix (ECM) deposition, which often severely destroys the patients’ physical appearance and function, decreasing their quality of life and delaying reintegration into society [1]. Previous studies have shown that many factors may significantly influence the pathogenesis of HTS. Other than inflammation, infection and mechanical force, genetic factors also play a vital role in increasing the susceptibility to HTS [2–6]. To our knowledge, the genome-wide association study (GWAS) that involved the largest sample size of HTS to date was the one reported by R.F. Sood. et al. [7]. They successfully collected complete genotypic and clinical data from 538 patients mainly involved the white people who were identified with ﻿a common intronic variant in the CUB and Sushi multiple domains 1 (CSMD1) gene related to the severity of postburn HTS [7]. However, further research has not yet been conducted to verify this finding in other HTS patients and to determine the underlying mechanism.

CSMD1 gene, mapping to human chromosomal region 8p23, encodes a transmembrane protein with an extracellular region containing 14 CUB and 28 sushi domains, a transmembrane domain and a cytoplasmic domain with a putative tyrosine phosphorylation site [8, 9]. The loss of CSMD1 ﻿has been found to be associated with enhanced cell proliferation, migration and poor prognosis in head and neck squamous cell carcinoma (HNSCCs), lung squamous cell carcinoma (SCCs), melanoma, and breast cancer, suggesting its role as a tumor suppresser [9–11]. However, no targeted drugs directly targeting the CSMD1 have been designed.

MicroRNAs (miRNAs) are a set of small non-coding RNAs less than 22 nucleotides, and usually negatively regulate the target mRNAs by binding to their 3′ -untranslated regions (3′-UTR) [12]. Therefore, we hope to identify the miRNAs that are able to interact with the CSMD1 as new targets for regulating CSMD1, which might be helpful in developing effective targeted drugs for HTS.

In this study, we verified significantly decreased expression of CSMD1 in both HTS tissue and HTS derived fibroblasts. The loss-of-function experiments were designed to explore the influence of CSMD1 knockdown on fibroblasts. Furthermore, databases were searched to identify microRNAs that could negatively regulate CSMD1, and was reconfirmed using luciferase reporter assay. Finally, RNA-sequencing (RNA-seq) was conducted to explore the downstream regulatory signaling pathway. Taken together, these observations have provided further evidence for the function of CSMD1particularly in the development of HTS, indicating a potential therapeutic target that may be useful for treatment of HTS.

## Results

### CSMD1 was down-regulated in hypertrophic scar tissue and HTS-derived fibroblasts

**Fig. 1:**
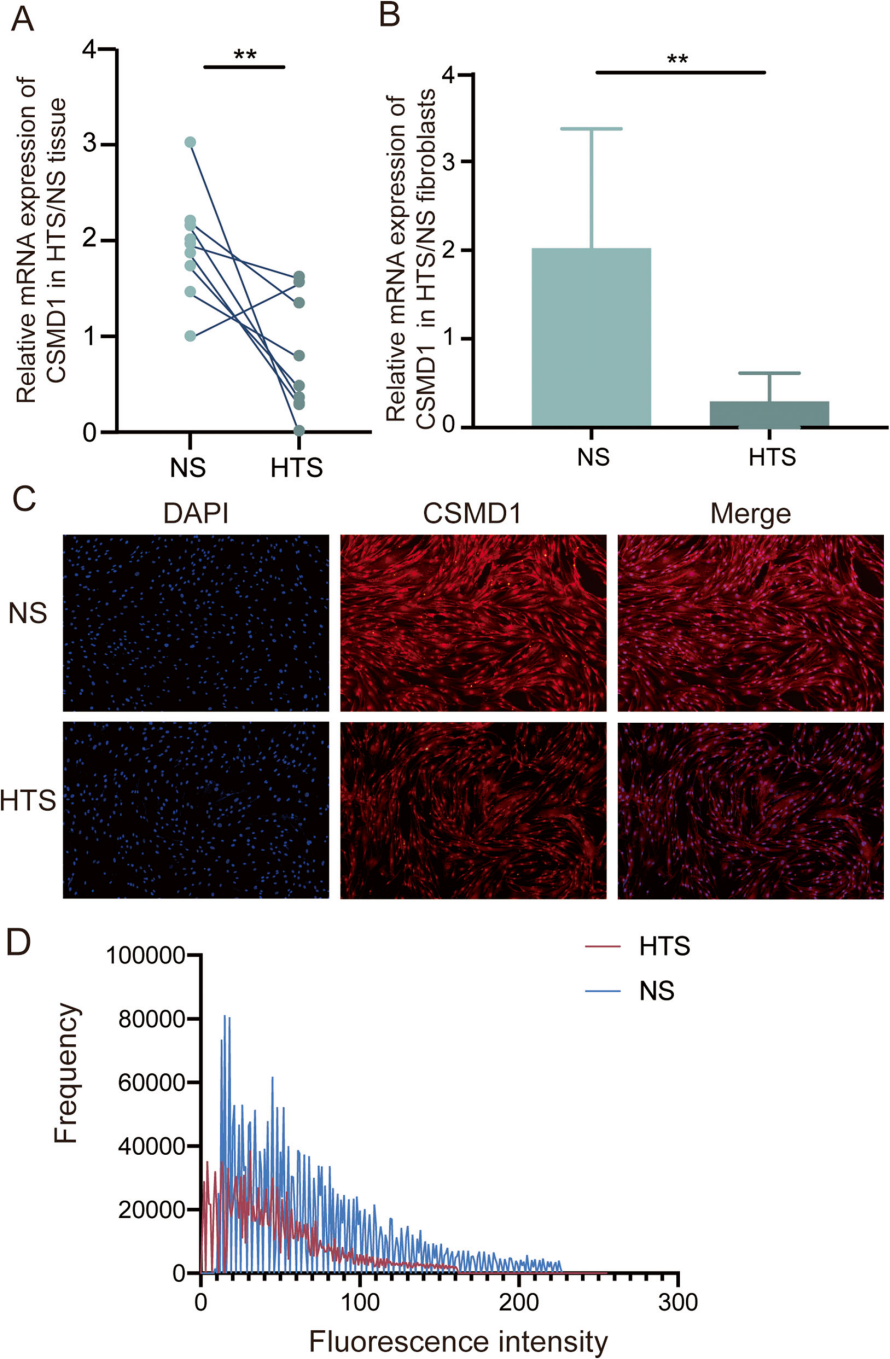
CSMD1 was down-regulated in hypertrophic scar tissue and HTS-derived fibroblasts. (**A**) The CSMD1 mRNA level in HTS tissue (*n* = 9) was significantly lower than that in the paired adjacent NS tissue (*n =* 9), **p < 0.01. (**B**) CSMD1 mRNA level in the HTS derived fibroblasts (*n* = 8) was significantly lower than that in NS derived fibroblasts (*n* = 2), *p < 0.05. (**C**) Immunofluorescence revealed lower expression of CSMD1 protein in the human HTS derived fibroblasts. Scale bar: 200 μm. (**D**) Histogram showing fluorescence intensity of CSMD1 in the HTS/NS derived fibroblasts from the IF photos taken and analyzed using the NIS-Elements D software

First, we explored the CSMD1 mRNA level in both human hypertrophic scar (HTS) tissue and the adjacent normal skin (NS) tissue. qRT-PCR result showed significantly down-regulated mRNA level of CSMD1 in the HTS tissue compared to the paired NS tissue (Fig. 1A). Similar downregulation of CSMD1 mRNA level was also found in the HTS and NS derived primary fibroblasts (Fig. 1B). Finally, IF confirmed the significantly down-regulated expression of CSMD1 on the protein level in HTS derived fibroblasts (Fig. 1C-D).

### Knockdown of CSMD1 promoted cell migration and fibronectin1 (FN1) secretion in fibroblasts

**Fig. 2:**
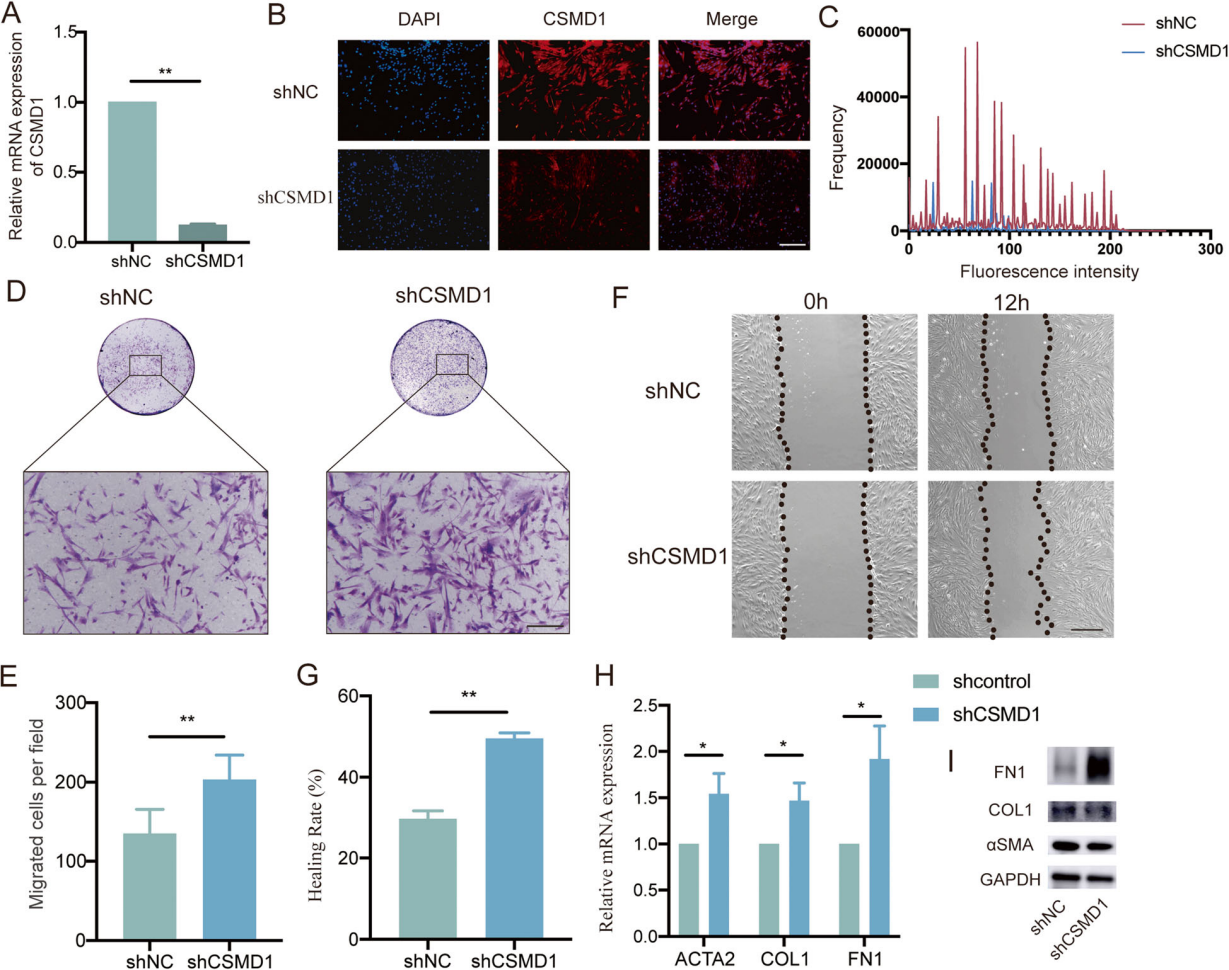
Knockdown of CSMD1 promoted cell migration and FN1 secretion in fibroblasts. (**A**) qRT-PCR was performed to compare the CSMD1 mRNA expression in fibroblasts transfected with Lenti-shRNA-CSMD1 (shCSMD1) and Lenti-GFP (shNC). All experiments were performed in triplicate and the data were shown as mean ± SD, *p < 0.05. (**B**) Immunofluorescence was performed to confirm the knockdown of CSMD1 protein expression in the CSMD1-silenced fibroblasts. Scale bar: 200 μm. (**C**) Histogram showing fluorescence intensity of CSMD1 in the shNC and shCSMD1 fibroblasts from the IF photos taken and analyzed using the NIS-Elements D software. (**D**-**E**) Transwell assays were performed to detect the migration of CSMD1-silenced fibroblasts. Quantification of numbers of migrated cells per field was presented as mean ± SD from three independent experiments in the right panel. Scale bar: 200 μm, **p < 0.01. (**F**-**G**) Wound healing assays were performed to detect the migration of CSMD1-silenced fibroblasts. The wound area at 0 h was set as 100%. Quantification of the healing rate was presented as mean ± SD from three independent experiments in the right panel. Scale bar: 200 μm, **p < 0.01. (**H**-**I**) qRT-PCR and western blot were performed to measure the ACTA2, COL1 and FN1 mRNA and protein levels respectively in the shNC and shCSMD1 fibroblasts. The results showed significantly upregulated mRNA levels of ACTA2, COL1 and FN1 upon CSMD1 knockdown, while only increased expression of FN1 at the protein level. *p < 0.05

To understand the biological effects of CSMD1 in fibroblasts, loss-of-function approach in CCD1064Sk cell line was conducted. After transfecting the lentiviruses with short hairpin sequences for CSMD1 (shCSMD1), both the mRNA and protein levels of CSMD1 were significantly reduced as compared to the negative control group (shNC) (Fig. 2A, B). Transwell assays revealed increased migratory activity of fibroblasts through the 8 μm pores upon CSMD1 knockdown (Fig. 2C). This result was further confirmed with wound healing assays that showed increased healing rate in the shCSMD1 group (Fig. 2D). qRT-PCR results revealed significantly upregulated mRNA levels of actin alpha 2, smooth muscle (ACTA2, αSMA), collagen1 (COL1) and FN1 (Fig. 2H) upon the knockdown of CSMD1. Western blot showed no obvious change in the protein level of αSMA and COL1, but revealed significantly upregulated protein level of FN1 in the shCSMD1 treated fibroblasts (Fig. 2I). Taken together, the loss of CSMD1 promoted the migration and fibronectin secretion in fibroblasts in vitro.

### MiR-190a-3p suppressed the expression of CSMD1 by targeting the 3′-UTR of CSMD1 mRNA

**Fig. 3:**
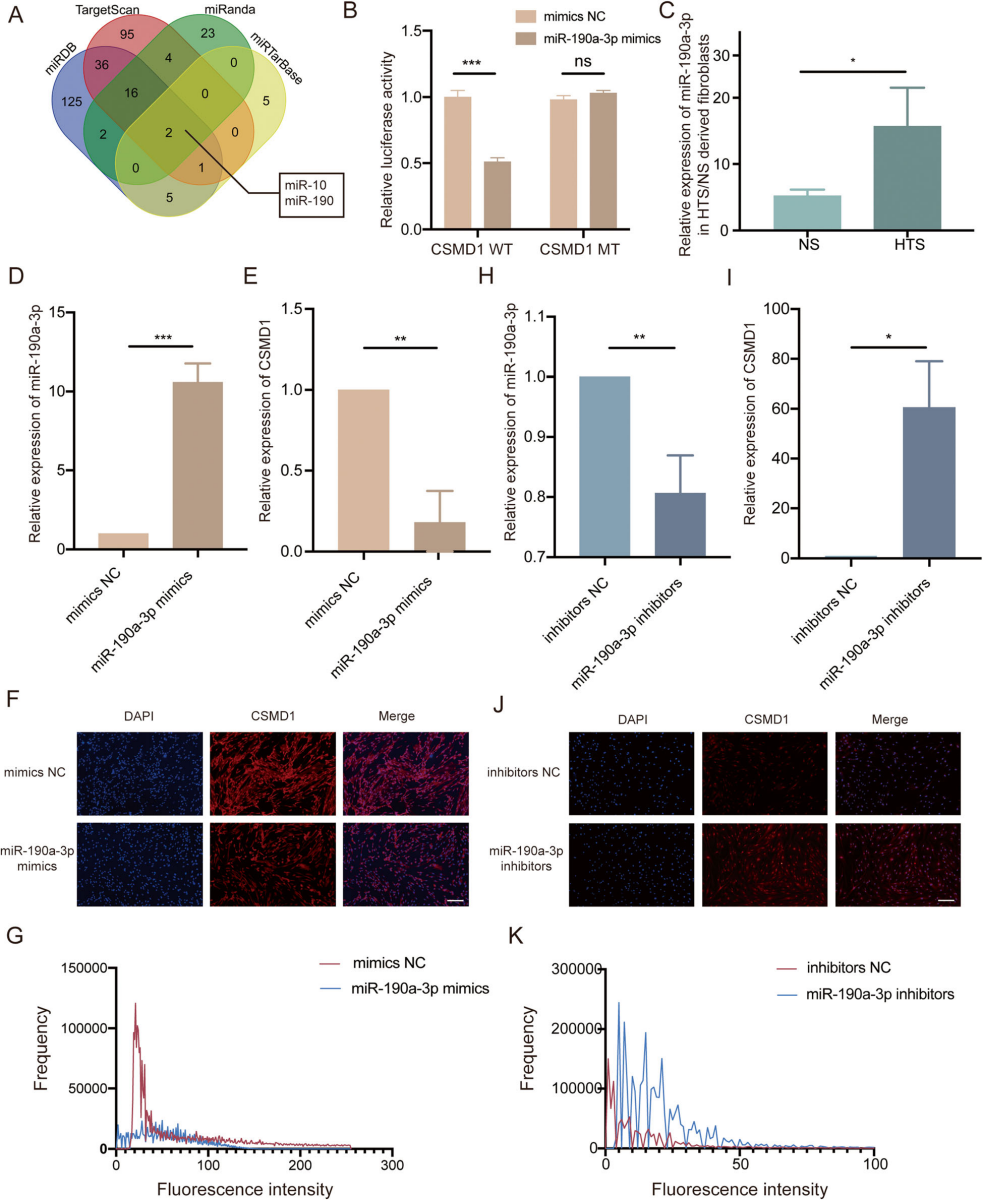
MiR-190a-3p suppressed the expression of CSMD1 by targeting the 3′-UTR of CSMD1 mRNA. (**A**) Two candidate microRNA families (miR-10 and miR-190) that target the CSMD1 gene were identified in four databases (miRDB, TargetScan, miRanda and miRTarBase) and are listed in Table 1. (**B**) Luciferase reporter assays demonstrated that miR-190a-3p mimics could significantly reduce the luciferase activity of CSMD1 in the wild type (WT) group, whereas the CSMD1 mutational type (MT) group was not affected, indicating that the direct binding of miR-190a-3p to the CSMD1–3′-UTR. ns, no significance, ***p < 0.001. (**C**) qRT-PCR confirmed comparatively higher expression of miR-190a-3p in HTS derived fibroblasts (*n* = 4) than in NS derived fibroblasts (*n* = 3), *p < 0.05. (**D**, **H**) qRT-PCR was performed to confirm successful overexpression/knockdown of miR-190a-3p using miR-190a-3p mimics/inhibitors respectively. Mimics NC/inhibitors NC were served as negative controls. All experiments were performed in triplicate and the data were shown as mean ± SD. **p < 0.01, ***p < 0.001. (**E**, **I**) qRT-PCR was performed to detect the CSMD1 mRNA level in fibroblasts treated with miR-190a-3p mimics/inhibitors and their negative controls. All experiments were performed in triplicate and the data were shown as mean ± SD. *p < 0.05, **p < 0.01. (**F**, **J**) Immunofluorescence was performed to confirm successful down-regulation/up-regulation of CSMD1 protein expression in fibroblasts treated with miR-190a-3p mimics/inhibitors. Scale bar: 200 μm. (**G**) Histogram showing fluorescence intensity of CSMD1 in the mimics NC and miR-190a-3p mimics/inhibitors-treated fibroblasts from the IF photos taken and analyzed using the NIS-Elements D software

Four universally used databases (miRDB, TargetScan, miRanda and miRTarBase) were searched to explore the candidate microRNA targeting CSMD1 gene. In order to eliminate the influence caused the change of microRNA names and the accuracy variations of databases, the subtypes ‘3p’ and ‘5p’ were not taken into consideration during the initial screening. As a result, two candidate microRNA families (miR-10 and miR-190) were identified in four databases (Fig. 3A). In these two families, four types of microRNA: miR-10b-5p, miR-10a-5p, miR-190a-5p and miR-190a-3p were predicted to interact with CSMD1 gene, and among which miR-190a-3p owned the largest number of possible binding sites and ranked first in the Target Score of miRDB with CSMD1 (Table 1).

**Table 1:** Description of candidate microRNAs targeting CSMD1 gene in miRDB

miRNA name	Target score	Number of potential binding sites	Seed location
miR-10a-5p	86	2	707, 2158
miR-10b-5p	86	2	707, 2158
miR-190a-5p	53	2	2218, 2369
miR-190a-3p	100	9	733, 2183, 2185, 2197, 2199, 2201, 2203, 2205, 2207

To confirm whether there was a direct interaction between miR-190a-3p and CSMD1, luciferase reporter assay was first performed. The results showed miR-190a-3p mimics significantly reduced the luciferase activity of CSMD1 in the wild type (WT) group, suggesting that CSMD1–3′-UTR could be a direct target of miR-190a-3p (Fig. 3B). Meanwhile, qRT-PCR showed an expressive abundance of miR-190a-3p in the HTS fibroblasts as compared to the adjacent NS fibroblasts normalized by U6 (Fig. 3C). qRT-PCR showed successful miR-190a-3p was achieved by transfecting miR-190a-3p mimics (Fig. 3D). Meanwhile, qRT-PCR and IF demonstrated that CSMD1 mRNA and protein levels were down-regulated in the miR-190a-3p mimics group (Fig. 3E-G), which further proved the interaction between them. MiR-190a-3p was down-regulated in fibroblasts treated with miR-190a-3p inhibitors (Fig. 3H). Meanwhile, mRNA and protein levels of CSMD1 were notably elevated (Fig. 3I-K). Collectively, we found that miR-190a-3p played a vital role in regulating the expression of CSMD1.

### The overexpression/ knockdown of miR-190a-3p promoted/ inhibited cell migration and FN1 secretion in fibroblasts

**Fig. 4:**
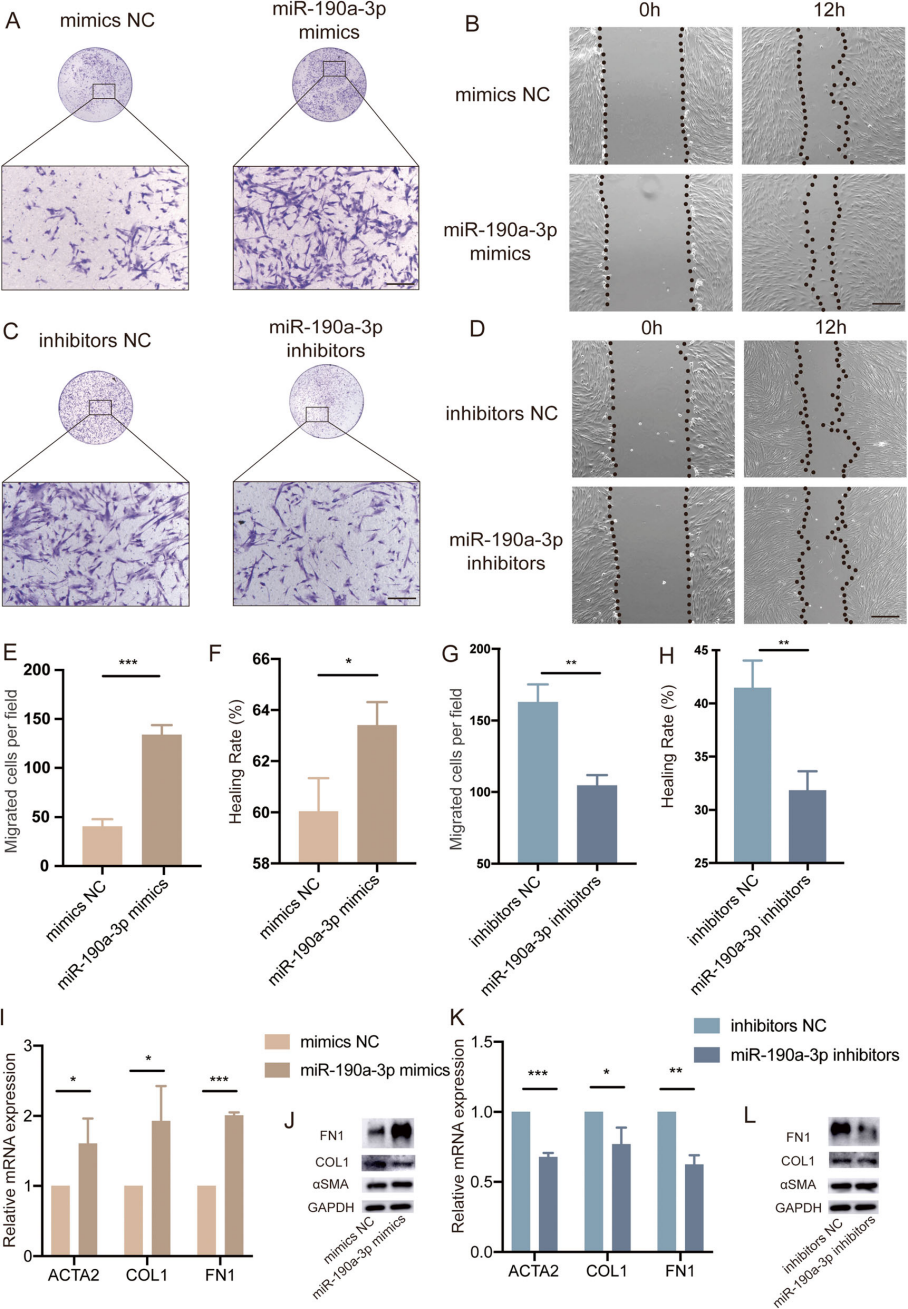
The overexpression/ knockdown of miR-190a-3p promoted/ inhibited cell migration and FN1 secretion in fibroblasts. (**A**, **E**) Transwell assay was performed to detect the migratory ability of the miR-190a-3p mimics-treated fibroblasts and was compared to negative control. Quantification of numbers of migrated cells per field was presented as mean ± SD from three independent experiments in panel E. Scale bar: 200 μm, ***p < 0.001. (**B**, **F**) Wound healing assay was performed to detect the migratory ability of the miR-190a-3p mimics-treated fibroblasts and was compared to negative control. The wound area at 0 h was set as 100%. Quantification of the healing rate from the three independent experiments in panel F was shown as mean ± SD. Scale bar: 200 μm, *p < 0.05. (**C**, **G**) Transwell assay was performed to detect the migratory ability of the miR-190a-3p inhibitors-treated fibroblasts and was compared to negative control. Quantification of numbers of migrated cells per field from three independent experiments in panel G was presented as mean ± SD. Scale bar: 200 μm, **p < 0.01. (**D**, **H**) Wound healing assay was performed to detect the migratory ability of miR-190a-3p mimics-treated fibroblasts and was compared to negative control. The wound area at 0 h was set as 100%. Quantification of the healing rate from three independent experiments in panel H was shown as mean ± SD. Scale bar: 200 μm, **p < 0.01. (**I**-**L**) qRT-PCR and western blot were performed to measure the ACTA2, COL1 and FN1 mRNA and protein levels in the mimics and inhibitors group. The results showed significantly upregulated ACTA2, COL1 and FN1 mRNA levels in the miR-190a-3p mimics group, while only increased expression of FN1 at the protein level; An opposite phenomenon was observed in the inhibitors group. *p < 0.05, **p < 0.01, ***p < 0.001

In order to evaluate the function of miR-190a-3p in fibroblasts, gain-and-loss-of-function approach was conducted in the CCD1064Sk cell line. Transwell assays showed that the significantly enhanced migration capacity of the miR-190a-3p mimics group as compared to the mimics NC group (Fig. 4A, E). Wound healing assays demonstrated enhanced healing rate in the miR-190a-3p mimics group (Fig. 4B, F). Whereas the transfection of miR-190a-3p inhibitors exerted the opposite effects. Both transwell and wound healing assays showed reduction in cell migration upon the knockdown of miR-190a-3p by miR-190a-3p inhibitors (Fig. 4C, D, G, H). qRT-PCR results revealed significantly upregulated mRNA levels of ACTA2, COL1 and FN1 in fibroblasts treated with miR-190a-3p mimics (Fig. 4I), while only FN1 had an increase at protein level (Fig. 4J). Oppositely, ACTA2, COL1 and FN1 mRNA levels were significantly downregulated in miR-190a-3p inhibitors group (Fig. 4K), while only FN1 expression decreased at the protein level (Fig. 4L). Taken together, miR-190a-3p promoted the migratory ability and fibronectin secretion in fibroblasts, probably through down-regulation of CSMD1 expression in HTS.

### Transcription profiling identified an activation of JAK/STAT signaling pathway in the CSMD1-silenced fibroblasts

**Fig. 5:**
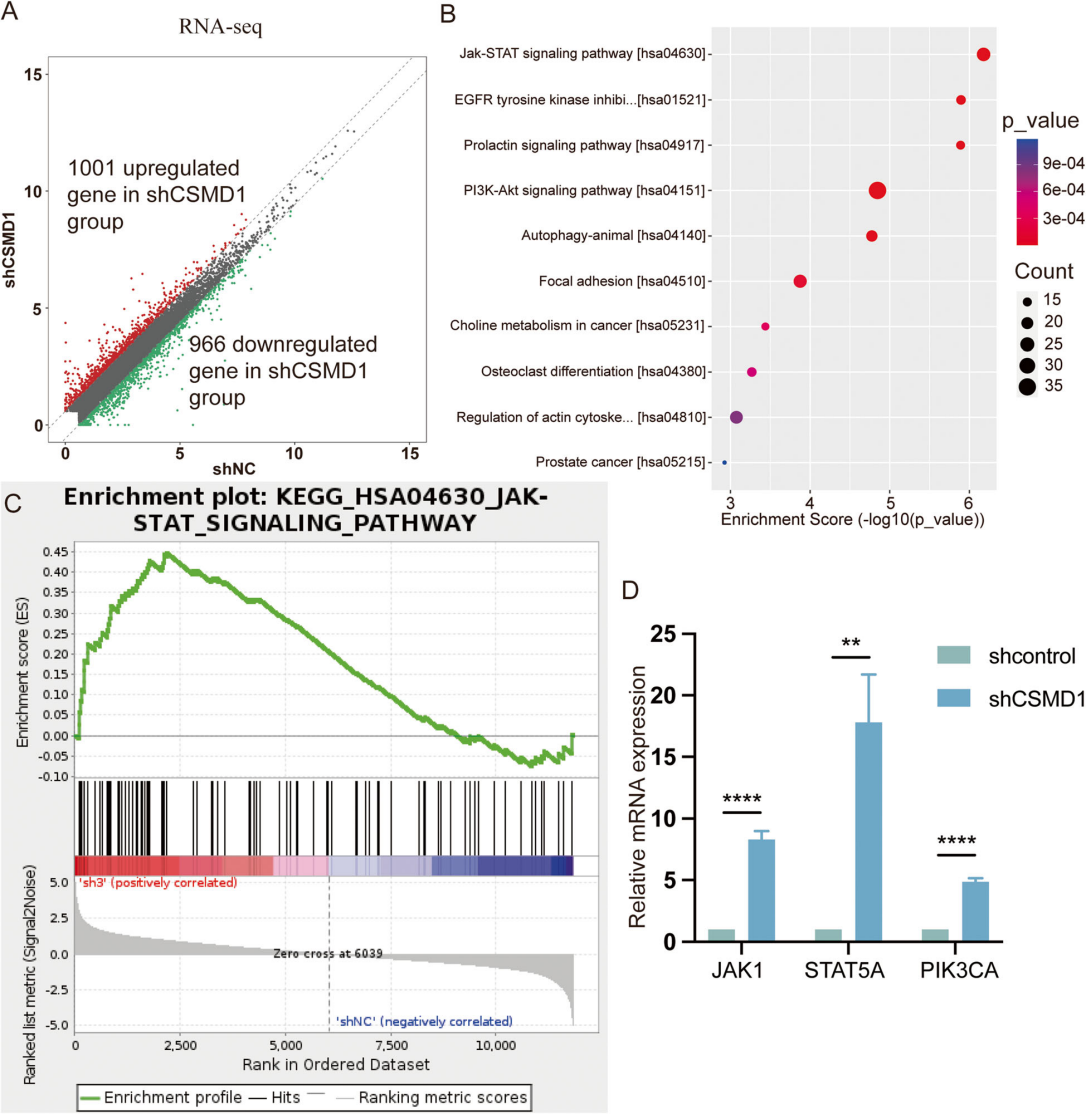
Transcription profiling identified an activation of JAK/STAT signaling pathway in the CSMD1-silenced fibroblasts. (**A**) Scatter plots of differentially expressed genes. 1001 genes were significantly up-regulated (red) while 966 genes were significantly down-regulated (green). Fold change> 1.5, *p* < 0.05. (**B**, **C**) Both Kyoto Encyclopedia of Genes and Genomes analysis and Gene Set Enrichment analysis revealed that JAK/STAT signaling pathway was the most significantly up-regulated pathway identified upon CSMD1 silencing. (**D**) qRT-PCR was performed to detect mRNA levels of the three essential genes in the JAK/STAT signaling pathway in fibroblasts treated with Lenti-shRNA-CSMD1 and was compared with its corresponding negative controls. JAK1, STAT5A and PIK3CA were significantly up-regulated in fibroblasts treated with Lenti-shRNA-CSMD1. All experiments were performed in triplicate and the data were shown as mean ± SD. **p < 0.01, ****p < 0.0001

To further explore the detailed downstream mechanism of the miR-190a-3p-CSMD1 axis, transcription profiling was performed on the CSMD1-silenced fibroblasts and its negative control. Results of RNA-seq assay displayed a total of 1967 differential expressed genes, and among which 1001 genes were up regulated while 966 genes were down regulated (Fig. 5A). Both Kyoto Encyclopedia of Genes and Genomes (KEGG) analysis and Gene Set Enrichment analysis (GSEA) revealed that JAK-STAT signaling pathway was the most significantly up-regulated pathway identified upon CSMD1 silencing (Fig. 5B, C). To further verify the results of RNA-seq assay, mRNA levels of the three essential genes in JAK/STAT signaling pathway, namely Janus kinase 1 (JAK1), signal transducer and activator of transcription 5A (STAT5A) and phosphatidylinositol-4,5-bisphosphate 3-kinase catalytic subunit alpha (PIK3CA),, were detected using qRT-PCR in fibroblasts treated with Lenti-shRNA-CSMD1. JAK1, STAT5A and PIK3CA were discovered to be significantly up-regulated in shCSMD1, which was consistent with the previous RNA-seq results (Fig. 5D).

## Discussion

As is known, cutaneous pathological scars includes hypertrophic scar and keloid. Increasing evidence showed that ﻿genetic susceptibility plays an important role in the occurrence and development of pathological scars [6, 13, 14]. In 2004, Marneros AG et al. performed genome scans and identified two susceptibility loci on chromosomes 2q23 and 7p11 [15]. Another microarray study revealed that keloid-prone patients exhibited alteration of caspase 6 and 14 transcripts [16]. In 2010, Nakashima M et al. introduced a multi-stage GWAS that includes 824 keloid cases and 3205 controls in the Japanese population, and identified four SNP loci in﻿ three chromosomal regions: 1q41, 3q22.3–23 and 15q21.3, that were closely associated with keloid [17]. GWAS conducted by R.F. Sood. et al. on the other hand, identified a common variants mapping near the CSMD1 gene locating on chromosome 8p23, is closely associated with the severity of postburn HTS [7]. From the experimental results above, there is no reported susceptibility gene to date specifically for pathological scars. Hence, we selected the pivotal gene CSMD1 from the postburn HTS GWAS study for further research, as patients with postburn scars make up the majority of patients in our clinic.

CSMD1 plays a vital role in multiple biological processes, and studies have shown that the mutations, deletions or methylation of CSMD1 were associated with wide range of diseases. Specifically, mutations in CSMD1 resulted in infertility problem both in males and females through ﻿complement-related processes [18]; the loss of CSMD1 could be found in head and neck squamous cell carcinoma (HNSCCs), lung squamous cell carcinoma (SCCs), melanoma, and breast cancer [9–11]; downregulation of CSMD1 mRNA expression by methylation of the CpG island in its promotor region was critical in the progression of hepatocellular carcinoma [19]. CSMD1 was first identified as a critical gene in a study which was conducted among predominantly non-Hispanic white, with one of the variants being associated with postburn HTS [7]. However, since then no further study has been conducted to assess and to further prove the effect of CSMD1 on hypertrophic scars. Therefore, we hypothesized that CSMD1 might play a decisive role in the occurrence and development of HTS.

For the first time, we intend to verify such results in the HTS tissues and fibroblasts specifically from the Chinese patients which maybe more prone to scarring, and found that the expression of CSMD1 was downregulated in the HTS compared to NS tissues and fibroblasts. As a potential tumor suppressor gene, the loss of CSMD1 impaired the morphology of mammary duct and enhanced cell proliferation, migration and invasion in breast cancer [9]. Similarly, Tang et al. found that CSMD1 positive cells displayed relatively weak proliferative and migratory capacity [10]. In our study, migratory activity of the fibroblast was enhanced evidently upon CSMD1 knock down, without affecting their proliferation capacity (Fig. S1), indicating that the effect of CSMD1 on fibroblast migration was not the consequences of altered cell growth, which might also be the main distinction between HTS and cancers.

Despite its relatively short length, miRNAs almost participate in all biological processes and have critical regulatory effect [20]. A number of studies have emphasized the importance of miRNAs in the development of HTS: miR-519d was confirmed to reduce proliferation and induce apoptosis in HTS fibroblasts [21]; miR-494 decreased HTS formation by targeting PTEN through PI3K/AKT signaling pathway [22]; miR-31-5p, promoted fibroblast proliferation, invasion and excessive extracellular matrix (ECM) deposition through activating the HIF-1α pathway [23]. Additionally, miRNAs might provide novel therapeutic targets for treatment of diseases including HTS [24]. For instance, it has been proven that miR-29 functions as a negative regulator in skin fibrosis and its mimic (Remlarsen) has been confirmed as a reliable therapeutic option in fibrotic scar (HTS or keloid) in a double-blinded, placebo-randomized within-subject controlled clinical trial [25]. Thus, we sought to identify the miRNA that directly targets CSMD1, which might potentially impact the development of HTS.

Due to its well-known effect on mRNA down-regulation, miRNA that targets the CSMD1 have also been explored in several oncology studies. For instance, miRNA-10b that targets the CSMD1 acted as an oncogenic factor in human gastric cancer through nuclear factor-κB (NF-κB) pathway [26]. In hepatocellular carcinoma, miR-10b was also demonstrated to promote cell viability and invasion through targeting CSMD1 [27]. While the current studies focused on the miR-10b, we discovered other potential binding sites in CSMD1 for miR-190a-3p which seems advantageous in comparison to miR-10b according to miRDB, and the direct interaction was later confirmed by luciferase reporter assay. MiR-190a-3p and miR-190a-5p were both generated from the miR-190 strands [28]. Although numerous studies showed that miR-190a-5p played a contradictory role in oncogenesis and progression [28], only two studies discussed the function of miR-190a-3p. Long intergenic non-coding LINC00657, sponging miR-190a-3p, was confirmed to inhibit glioblastoma [29]. Similarly, circCDYL suppressed triple negative breast cancer by sponging miR-190a-3p, whereas the up-regulation of miR-190a-3p led to a reverse effect [30]. These observations were in consistence with our findings regarding the pro-migratory capacity of miR-190a-3p in fibroblasts.

Additionally, our research showed that the knockdown of CSMD1 activated the JAK/STAT signaling pathway. JAK/STAT pathway is a critical signaling pathway in multiple biological processes including proliferation and migration, especially in immunoregulation [31–33]. Four different mutations of JAK (JAK1, JAK2, JAK3, TYK2) and 7 different mutations of STAT (STAT1, STAT2, STAT3, STAT4, STAT5A, STAT5B, STAT6) function differently in various diseases [31]. Among them, JAK1 and STAT1 were proved to be involved in the proliferation and differentiation of human hypertrophic scar fibroblasts treated with connective tissue growth factor [34]. In our results, JAK1 and STAT5A were revealed as the activated subtypes in this pathway, indicating that the enhanced migration of skin fibroblasts was probably mediated by the JAK1/STAT5A.

The inhibitors targeting JAKs (Jakinibs) were used as a therapeutic strategy for immune and inflammatory diseases [35, 36]. Numerous clinical trials have been conducted and the selective Jakinibs had already been produced and studied in various diseases [36]. Oral JAK1 selective inhibitors, upadacitinib and filgotinib, were both reported to exert positive effect on active rheumatoid arthritis patients [37, 38]. Hence, targeted drugs that target critical cellular signaling pathways hold promising potential for the treatment of HTS in the future.

## Conclusions

In conclusion, our study demonstrated that miR-190a-3p directly down-regulated CSMD1, and promoted migratory activity and FN1 secretion in fibroblast. More researches are required to clarify the exact function and mechanism of CSMD1 in HTS, which will hopefully bring about new therapeutic approaches for HTS.

## Methods

### Cell line, clinical samples and ethics statement

Normal human skin fibroblast cell line (CCD­1064Sk, ATCC® CRL­ 2076TM) were purchased from American Type Culture Collection (ATCC), and cultured according to the instruction. Hypertrophic scars and normal skin samples were obtained from patients undergoing surgeries in the Department of Plastic and Reconstructive Surgery in Shanghai Ninth People’s Hospital, Shanghai Jiaotong University School of Medicine. All patients were well informed about the utilization of specimens and signed written informed consent forms. This study was approved by Shanghai Ninth People’s Hospital Ethics Committee Board, Shanghai Jiaotong University School of Medicine (Shanghai, China), and followed the ethical principles of the Declaration of Helsinki 1964.

### Cell culture and treatment

Isolation and culture of human HTS and NS fibroblasts were conducted as previously described. Specifically, specimens obtained during surgery were cut into 5 mm × 5 mm pieces and soaked in 0.3% dispase II (0.3 g/ml; Gibco, 17,105,041) at 4 °C for 12 h. Then, the epidermis was torn off and the dermis were minced and incubated in collagenase NB4 (3 mg/ml; Nordmark, S1745401) at 37 °C for 4 h to isolate the dermal fibroblasts. Primary fibroblasts were cultured in Dulbecco’s modified Eagle’s medium (DMEM, Gibco, USA) and CCD-1064Sk was cultured in Iscove’s modified Dulbecco’s medium (IMDM, Hyclone, USA), both containing 10% Fetal Bovine Serum (FBS, Gibco, USA) and 1%﻿ penicillin-streptomycin (Gibco, USA) and then incubated at 37 °C in a humidified atmosphere with 5% CO_2_.

### RNA/microRNA isolation and quantitative real-time PCR (qRT-PCR)

Total RNA was extracted using AxyPrep™ Multisource Total RNA Miniprep Kit (Axygen, USA) and reverse transcribed into cDNA using PrimeScript RT reagent Kit (Takara, Japan). Real-time PCR was performed with SYBR Premix EX Taq (Takara, Japan), using Glyceraldehyde-3-phosphate dehydrogenase (GAPDH) (Sangon Biotech, China) as the endogenous reference. ﻿MicroRNA was extracted using SanPrep Column microRNA Extraction Kit (Sangon Biotech, China), and reverse transcribed using miRNA First Strand cDNA Synthesis (Tailing Reaction) (Sangon Biotech, China). Real-time PCR was performed with MicroRNAs qPCR Kit (SYBR Green Method) (Sangon Biotech, China), using U6 nuclear small RNA (Sangon Biotech, China) as the endogenous reference. 2^-△△Ct^ method was used to calculate the relative expression levels. Specifically, in Figs. 1A, B and 3C, the expression level of one NS was defined as 1, and normalized expression levels were converted to fold changes. All experiments were conducted according to the manufacturer’s instructions. Primers used in this study are as follows:

CSMD1 forward (5′-CATAAGTTACAGCTGCATGGAC-3′),

CSMD1 reverse (5′-GAAACTTTTCCCACTAAGTCGC-3′),

ACTA2 forward (5′-AAAAGACAGCTACGTGGGTGA-3′),

ACTA2 reverse (5′-GCCATGTTCTATCGGGTACTTC-3′),

COL1 forward (5′-GCTTGGTCCACTTGCTTGAA-3′),

COL1 reverse (5′-TTTGGGAAGGAGTGGAGGG-3′),

FN1 forward (5′-CGGTGGCTGTCAGTCAAAG-3′),

FN1 reverse (5′-AAACCTCGGCTTCCTCCATAA-3′),

JAK1 forward (5′-CTTTGCCCTGTATGACGAGAAC-3′),

JAK1 reverse (5′-ACCTCATCCGGTAGTGGAGC-3′),

STAT5A forward (5′-GCAGAGTCCGTGACAGAGG-3′),

STAT5A reverse (5′-CCACAGGTAGGGACAGAGTCT-3′),

PIK3CA forward (5′-GAAACAAGACGACTTTGTGACCT-3′),

PIK3CA reverse (5′-CTTCACGGTTGCCTACTGGT-3′),

miR-190a-3p forward (5′-GCGCGCGCTATATATCAAACATATTCC-3′).

### Immunofluorescence (IF)

Fibroblasts were seeded in 48-well dishes and cultured until 80% confluence. After fixed in 4% paraformaldehyde at room temperature for 20 min, washed with PBS, permeabilized and blocked, cell samples were stained with anti-CSMD1 antibody (1:100; Abcam, ab198906) at 4 °C for 12 h. Then, cell samples were incubated with goat anti-rabbit secondary antibody, Alexa Fluor 594 (1:400; Cell Signaling Technology, 8889S). Nuclei were stained with DAPI. Images were collected with fluorescence microscope. All experiments were conducted according to the manufacturer’s instructions.

### Cell transfection

The short hairpin RNA (shRNA) targeting CSMD1 was synthetized and the recombinant lentiviral vectors (Lenti-shRNA-CSMD1) were generated by Zorin Co. Ltd. (Shanghai, China). The RNA interference target was 5′-GCATACAACCCACCTGCATTG-3′. ﻿Lentiviral vector carrying green fluorescent protein without target genes (Lenti-GFP) was constructed as negative control. CCD1064Sk cell line was transfected with Lenti-shRNA-CSMD1 (shCSMD1) and Lenti-GFP (shNC) respectively at MOI of 30 in 6-well dishes for 24 h, with the assistance of 6 μg/ml polybrene. 48 h after transfection, the transfection efficiencies were briefly evaluated by observing green fluorescence with a fluorescence microscope. Meanwhile, RNA samples were extracted from the transfected cells for qRT-PCR to further evaluate the knockdown of CSMD1 and protein levels were evaluated by IF.

MiR-190a-3p mimics/inhibitors and their negative controls were purchased from Genomeditech Co. Ltd. (Shanghai, China). CCD1064Sk cell line was transfected with them in 6-well dishes for 6 h, with the assistance of Lipofectamine 2000 Transfection Reagent (ThermoFisher, USA). 48 h after transfection, microRNA samples were extracted from the transfected cells for qRT-PCR to evaluate the overexpression or knockdown of miR-190a-3p. All experiments were conducted according to the manufacturer’s instructions.

### Luciferase reporter assay

Database miRDB (http://mirdb.org) was searched to predict the potential binding sites between miR-190a-3p and the 3′-untranslated regions (3’UTR) of CSMD1. MiR-190a-3p owned the largest number of possible binding sites and ranked first in the Target Score of miRDB with CSMD1. Therefore, luciferase reporter assays were carried out to confirm that miR-190a-3p directly targeted CSMD1–3′-UTR. Mutations were generated within CSMD1–3’UTR to disrupt the potential binding. Then, the wild type (CSMD1 WT), mutational type (CSMD1 MT) and UTR negative control (UTR NC) were cloned into pGL-CMV luciferase reporter plasmid, which was co-transfected with miR-190a-3p mimics and mimics negative control (mimics NC) using Lipofectamine 2000 Transfection Reagent (ThermoFisher, USA). At 48 h following the transfection, luciferase activity was detected using the Dual Luciferase Reporter Assay System (Promega, Madison, WI, USA) according to the manufacturer’s instructions.

### Transwell assay

A 24-well transwell system with polycarbonate filters (8 μm pores, Corning, USA) was used. The upper chamber contained 1 × 10^5^ cells suspended in 200 μl IMDM without FBS and the lower chamber contained 500 μl IMDM with 20% FBS. After 48 h of incubation at 37 °C, the cells were stained with 0.1% crystal violet. The cells in the upper chamber were removed, and the numbers of migrated cells were photographed and counted by randomly selecting 5 views.

### Wound healing assays

Cells were seeded into 6-well dishes and grown until 95% confluence. A sterilized 200 μl pipette tip was used to generate a scratch. After the debris was washed away, fresh serum-free IMDM was added and images of cell migrations were taken at 5 randomly selected views per well using a microscope at 0 h and 12 h respectively. The areas of scratch at 0 h were set as 100% and the healing rates of different groups at 12 h were compared.

### Western blot analysis

The protein samples were collected 72 h after respective treatment. Equal amounts of proteins were resolved on 10% SDS-polyacrylamide gel and transferred to PVDF membrane (Millipore, Bedford, MA, USA) for 2 to 3 h. The membranes were then incubated with primary antibody overnight at 4 °C. The membranes were then incubated with appropriate secondary antibody for 1 h at room temperature and then developed with ECL western blotting detection reagent (Millipore, Billerica, MA) following the manufacturer’s instruction. Primary antibodies used in this article are as follows: anti-GAPDH mouse monoclonal antibody-HRP conjugated (BE0034, Easybio, China), anti-αSMA (AF1032, Affinity, China), anti-FN1 (AF5335, Affinity, China).

### RNA-sequencing (RNA-seq)

Total RNA was extracted from the fibroblast cell line (CCD1064Sk) after the knockdown of CSMD1 and its negative control using TRIzol reagent (Invitrogen, USA). The mRNA levels of the unigenes identified using Illumina HiSeq 4000 and were normalized by the Fragments Per Kilobase of exon model per Million mapped reads (FPKM), and the log2-fold changes between two samples were tested statistically to determine whether an individual gene’s expression was altered significantly. We used the criteria of false discovery rate (FDR) < 0.01 and fold changes > 1.5 (*p* < 0.05) to identify the differentially expressed genes.

### Statistical analyses

Each experiment was replicated three times. Results are presented as means ± SD. Statistical differences among groups were assessed using a two-tailed Student’s *t* test or ANOVA. *P* < 0.05 was considered to be statistically significant.

## Supplementary Information

**Additional file 1.**

## Data Availability

The datasets analyzed during the current study are available from the corresponding author on reasonable request. The original data were deposited in the Gene Expression Omnibus database with series ID GSE176551.

## Abbreviations

CSMD1: CUB and Sushi multiple domains 1
HTS: Hypertrophic scar
shRNA: Short-hairpin RNA
miRNA: microRNA
ACTA2, αSMA: Actin alpha 2, smooth muscle
FN1: Fibronectin1
COL1: Collagen1
RNA-seq: RNA-sequencing
Jak-STAT signaling pathway: Janus kinase-signal transducer and activator of transcription signaling pathway
GWAS: Genome-wide association study
HNSCCs: Head and neck squamous cell carcinoma
SCCs: Squamous cell carcinoma
3′-UTR: 3′-untranslated regions
ECM: Excessive extracellular matrix
ATCC: American Type Culture Collection
GAPDH: Glyceraldehyde-3-phosphate dehydrogenase
IF: Immunofluorescence
WT: Wild type
MT: Mutational type
NC: Negative control
NS: Normal skin
KEGG: Kyoto Encyclopedia of Genes and Genomes
GSEA: Gene Set Enrichment analysis
PIK3CA: Phosphatidylinositol-4,5-bisphosphate 3-kinase catalytic subunit alpha
NF-κB: Nuclear factor-κB
