# Supplementary material for: Inhibition of CUB and sushi multiple domains 1 (CSMD1) expression by miRNA-190a-3p enhances hypertrophic scar-derived fibroblast migration in vitro
Source: BMC Genomics. 2021 Aug 12;22:613. doi: 10.1186/s12864-021-07920-8 (PMC8359300; doi:10.1186/s12864-021-07920-8)
Supplement: Supplementary file 1 — Additional file 1. [file 12864_2021_7920_MOESM1_ESM.docx]

**Title Page**

**Inhibition of CUB and Sushi multiple domains 1 (CSMD1) expression by miRNA-190a-3p enhances hypertrophic scar-derived fibroblast migration in vitro**

**Authors:**

Shuchen Gu^#^, Xin Huang^#^, Xiangwen Xu, Yunhan Liu, Yimin Khoong, Zewei Zhang, Haizhou Li, Yashan Gao, Tao Zan **^*^**

**Authors’ affiliations:**

Department of Plastic and Reconstructive Surgery, Shanghai Ninth People’s Hospital, Shanghai JiaoTong University School of Medicine, Shanghai, China, 200011

**^*^Corresponding Author:**

Tao Zan, MD, PhD.

Mail address: Department of Plastic and Reconstructive Surgery, The Ninth People’s Hospital, Shanghai Jiao Tong University School of Medicine, 639 Zhizaoju Road, Shanghai 200011, P.R. China.

Email: zantaodoctor@yahoo.com

Telephone/Fax: +86 21 63089567

^#^ **Co-first authors：**

Shuchen Gu and Xin Huang contributed equally to the article, and should be viewed as co-first authors.

**Supplementary materials**

**1. Materials and Methods**

**1.1 Cell viability assay (CCK-8 assay)**

Cell viability of fibroblasts was detected with CCK-8 Cell proliferation Assay Kit (Dojindo Laboratories, Mashiki, Japan). Briefly, fibroblasts were seeded in 96-plate at the density of 3000 cells per well with IMDM and were tested with CCK-8 reagent at 0h, 24h, 48h and 72h. The assay was carried out in triplicate and repeated with three independent pooled cell samples.

**1.2Flow cytometric analysis on cell cycle**

After transfected with lenti-shRNA-CSMD1 and negative control, fibroblasts were collected as single cell suspensions. The cell suspensions were washed with PBS twice and were then fixed with 70 % ethanol overnight. Propidium iodide single stain reagent (Beyotime Biotechnology, Shanghai, China) was added and incubated for 30 min. The stained fibroblasts were analyzed using a flow cytometric with the Cell Quest acquisition and analysis software program.

**2. Figures and figure legends**

**
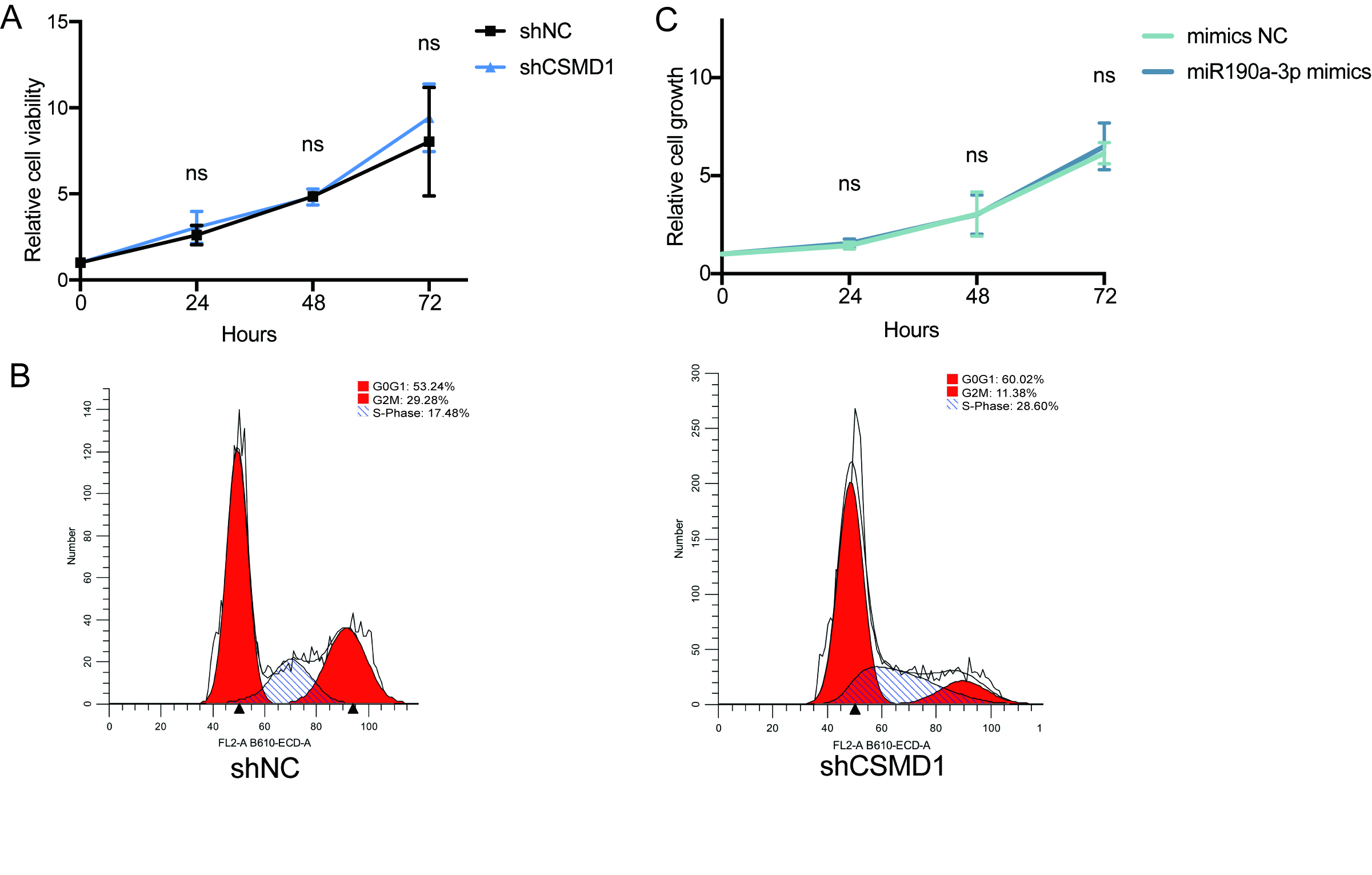
Figure S1.** **Knockdown of CSMD1 did not influence the proliferation of fibroblasts.**

(A) CCK-8 assays in shCSMD1 group and its negative control group showed that knockdown of CSMD1 did not significantly influence the proliferation of fibroblast. (B) Flow cytometric analysis demonstrated that knockdown of CSMD1 did not significantly affect the phase distribution of fibroblasts. (C) Similar to the knockdown of CSMD1, overexpression of miR-190a-3p in miR-190a-3p mimics group and its negative control group displayed no significant change in proliferation of fibroblasts in CCK-8 assays. ns, no significance.


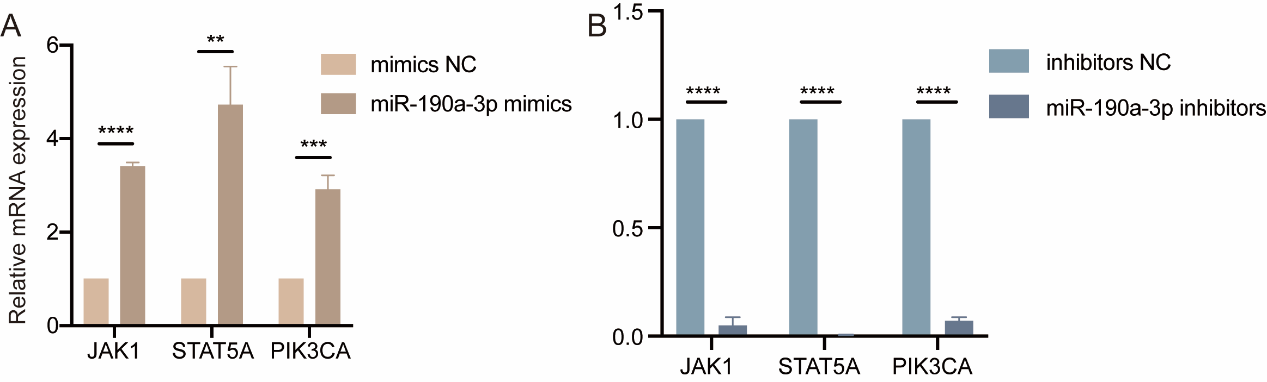


**Figure S2. miR-190a-3p regulated the mRNA expression of JAK1, STAT5A and PIK3CA.**

qRT-PCR was performed to detect the mRNA levels of three essential genes in JAK-STAT signaling pathway in fibroblasts treated with miR-190a-3p mimics and inhibitors with their corresponding negative controls. JAK1, STAT5A and PIK3CA were significantly up-regulated in fibroblasts treated with miR-190a-3p mimics (A), while down-regulated in those treated with miR-190a-3p inhibitors (B). All experiments were performed in triplicate and the data were shown as mean ± SD. ***p*<0.01, ****p*<0.001, *****p*<0.0001.


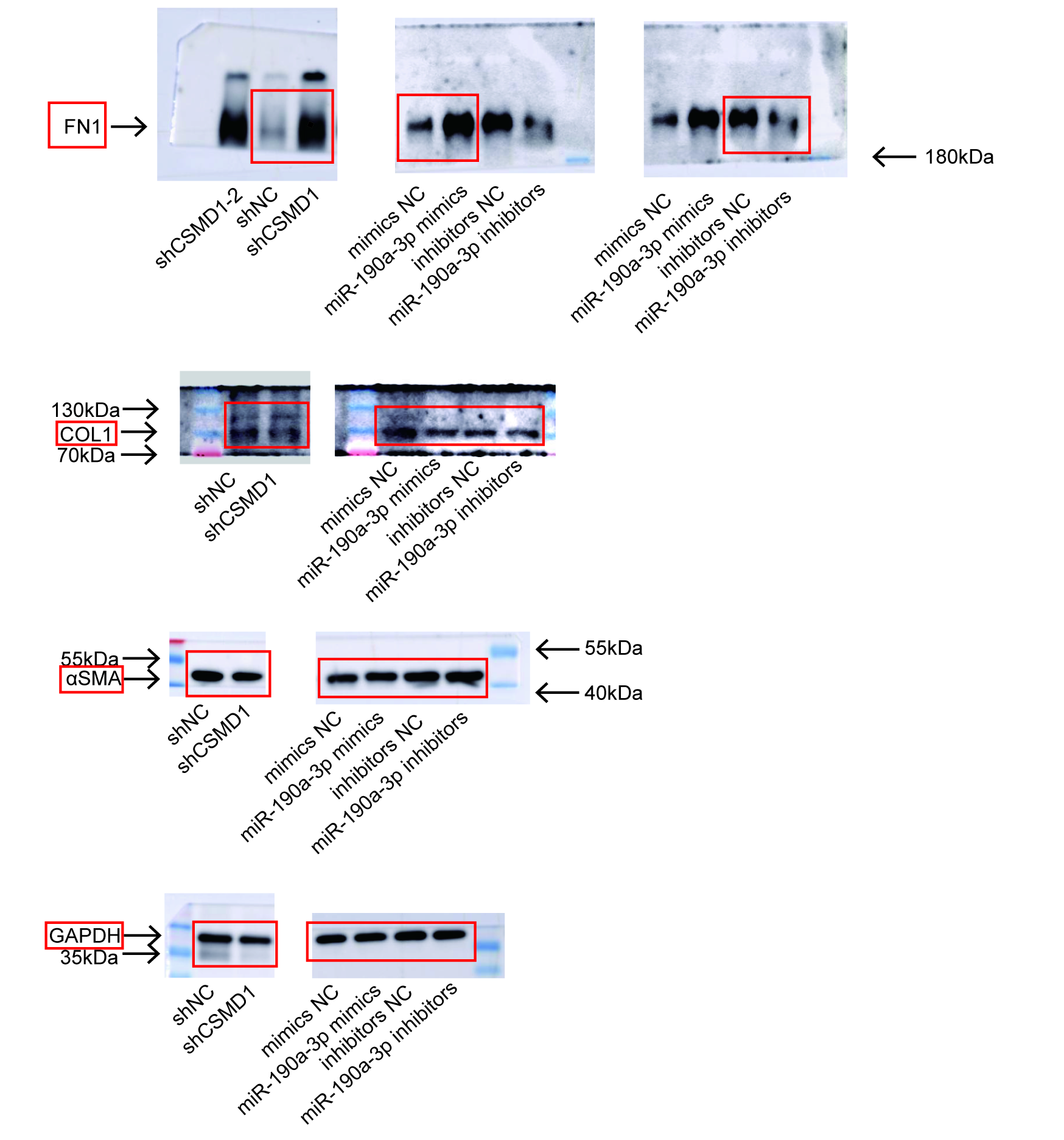


Figure S3. The original pictures of Western Blot. The bands in red boxes were those shown in the manuscript. Markers: thermo #26616.
